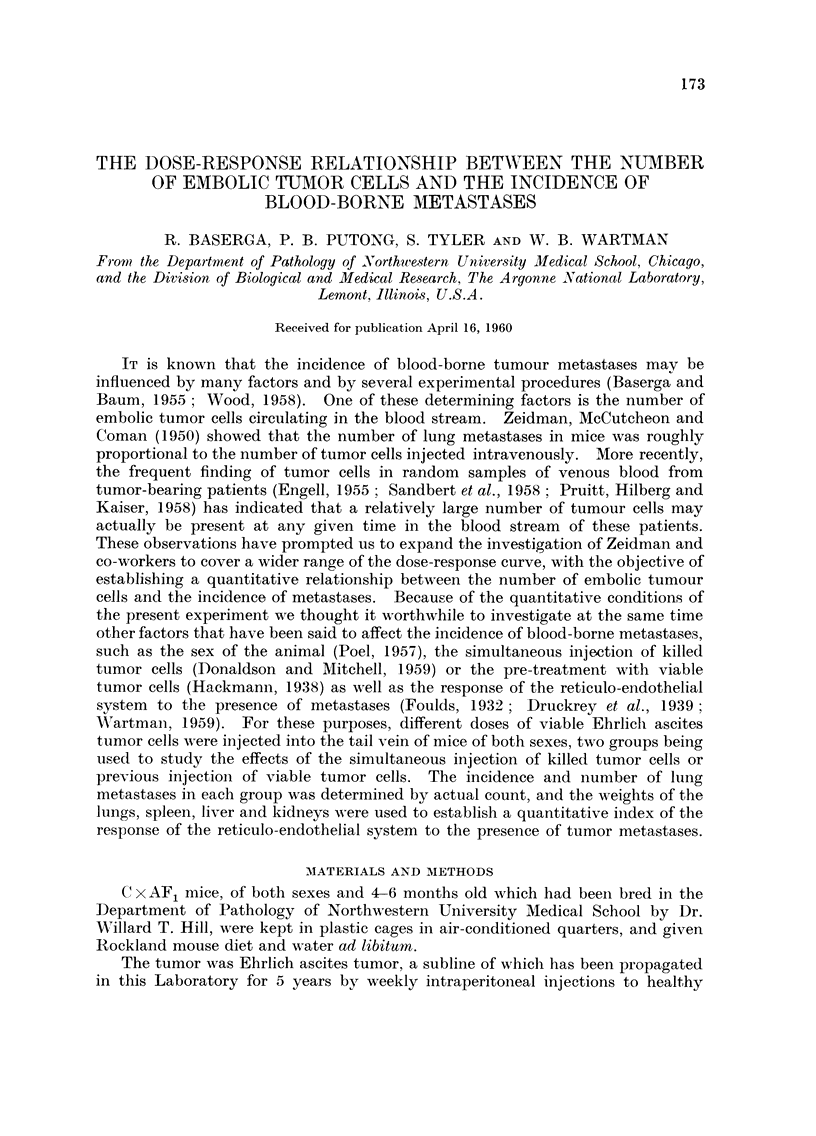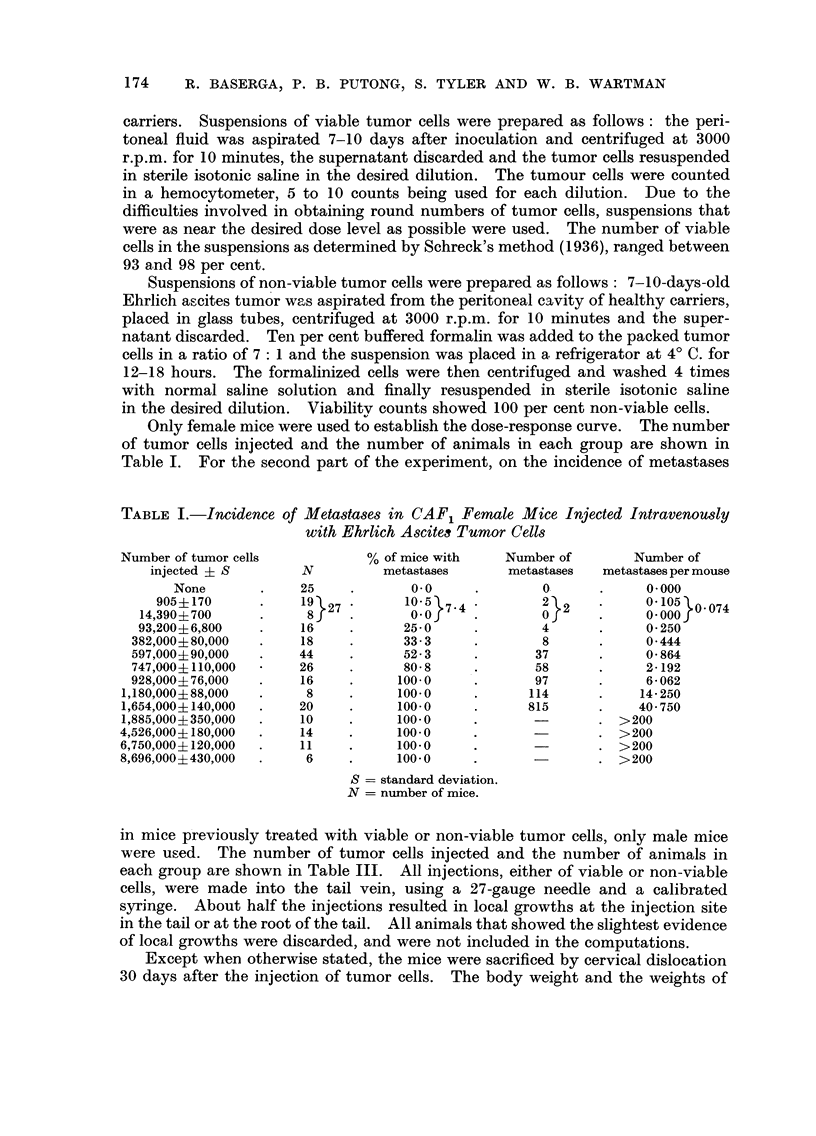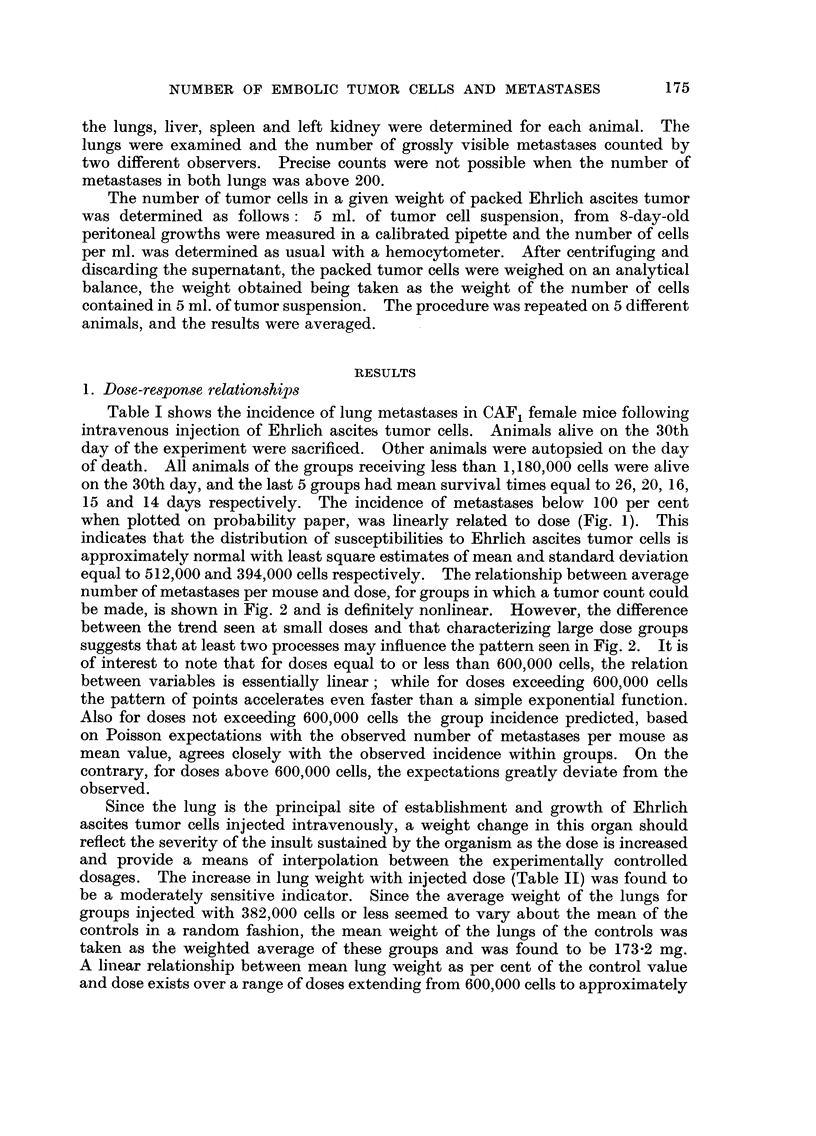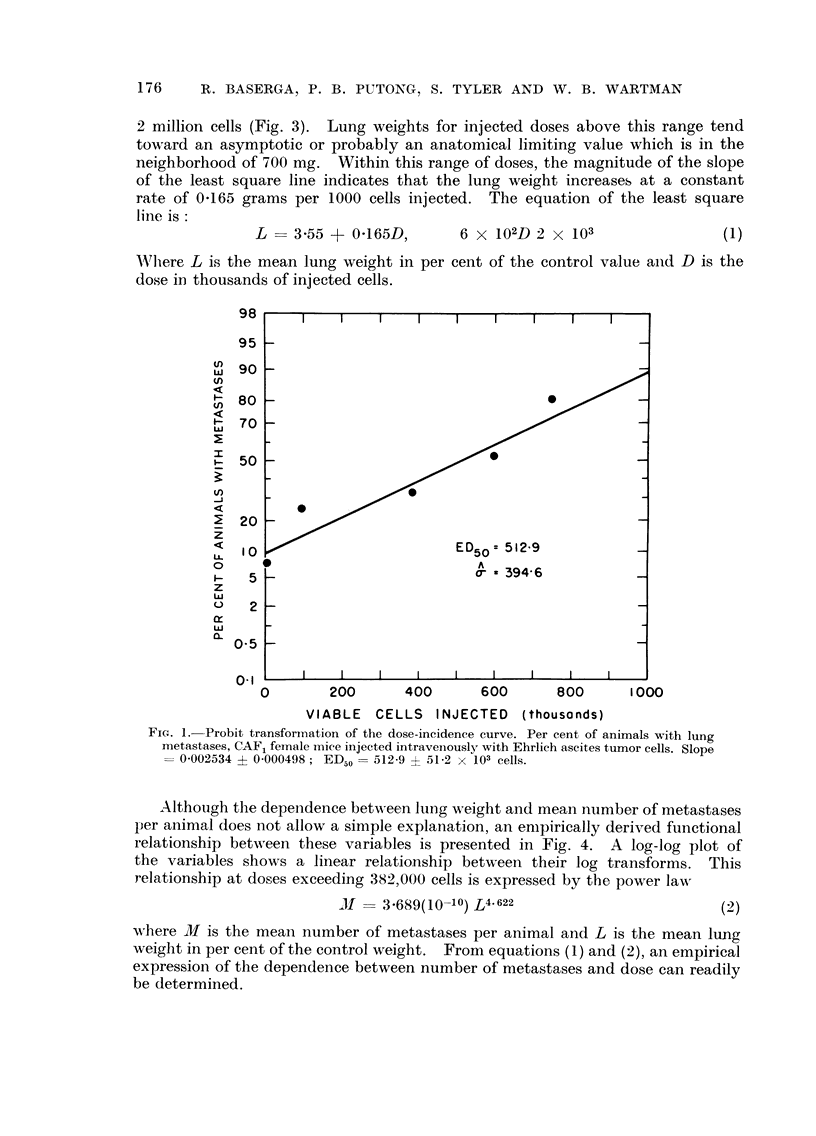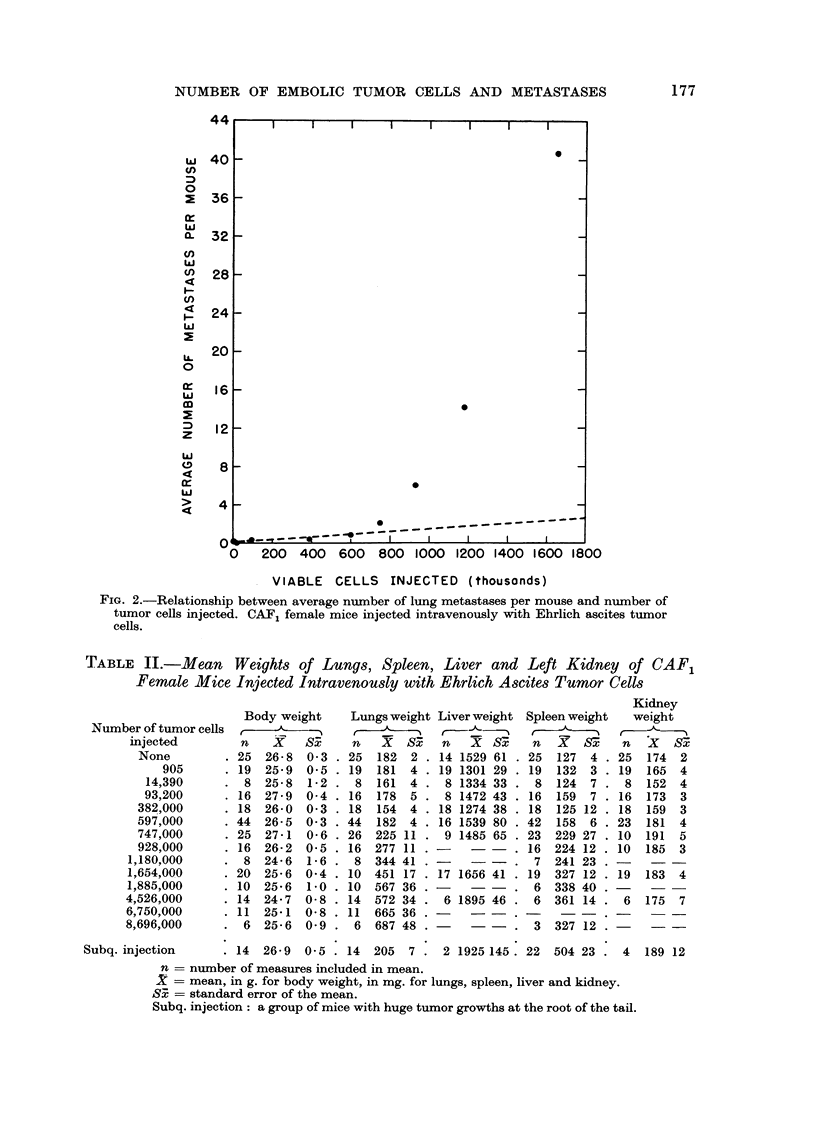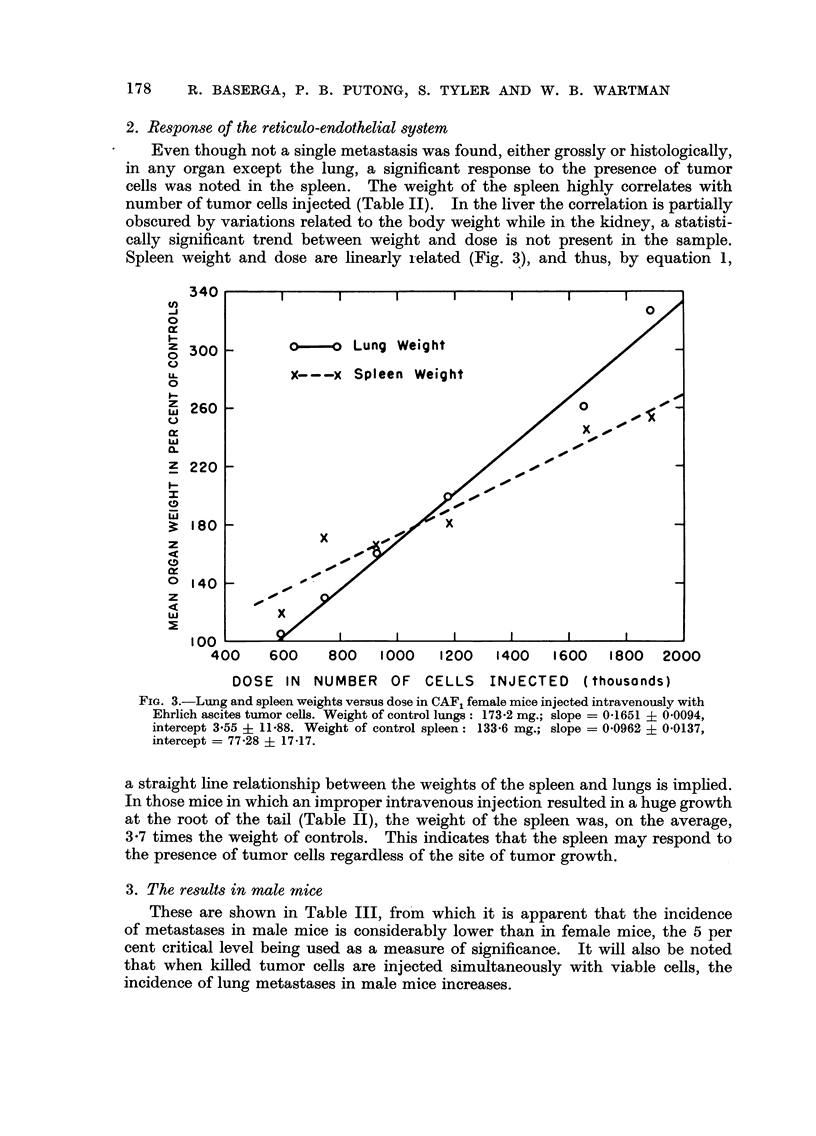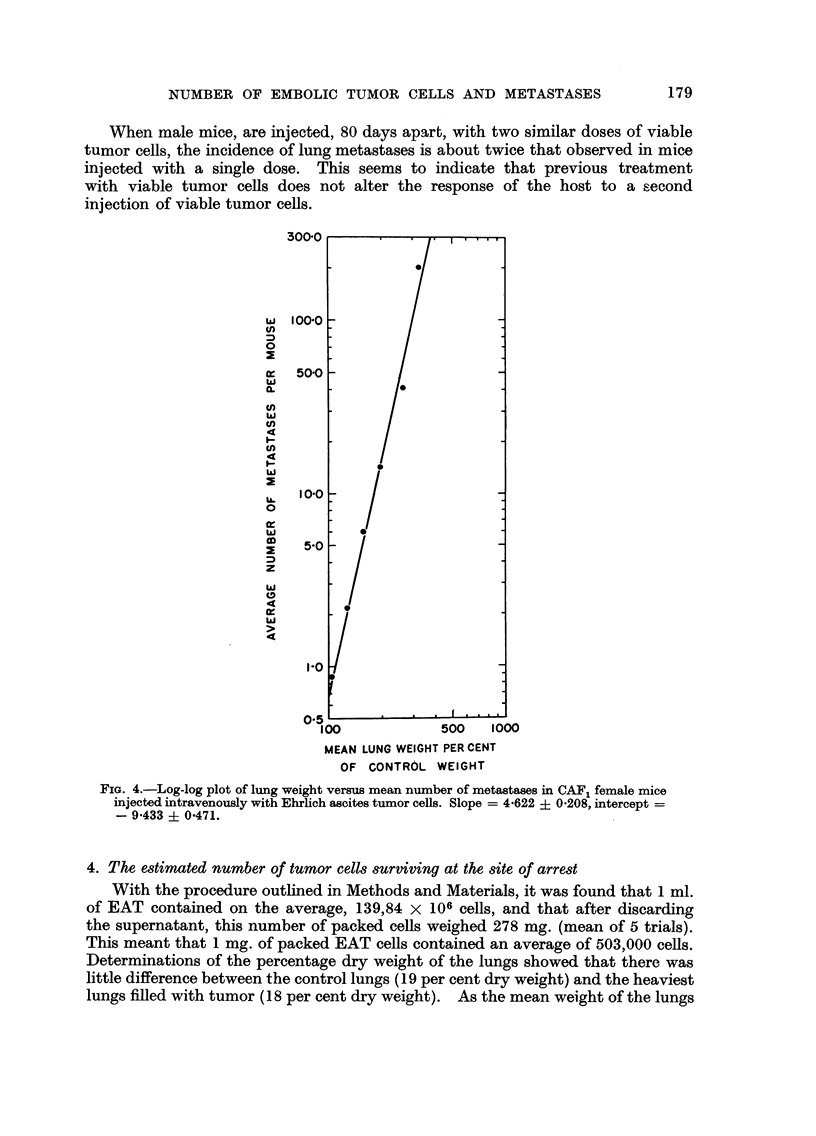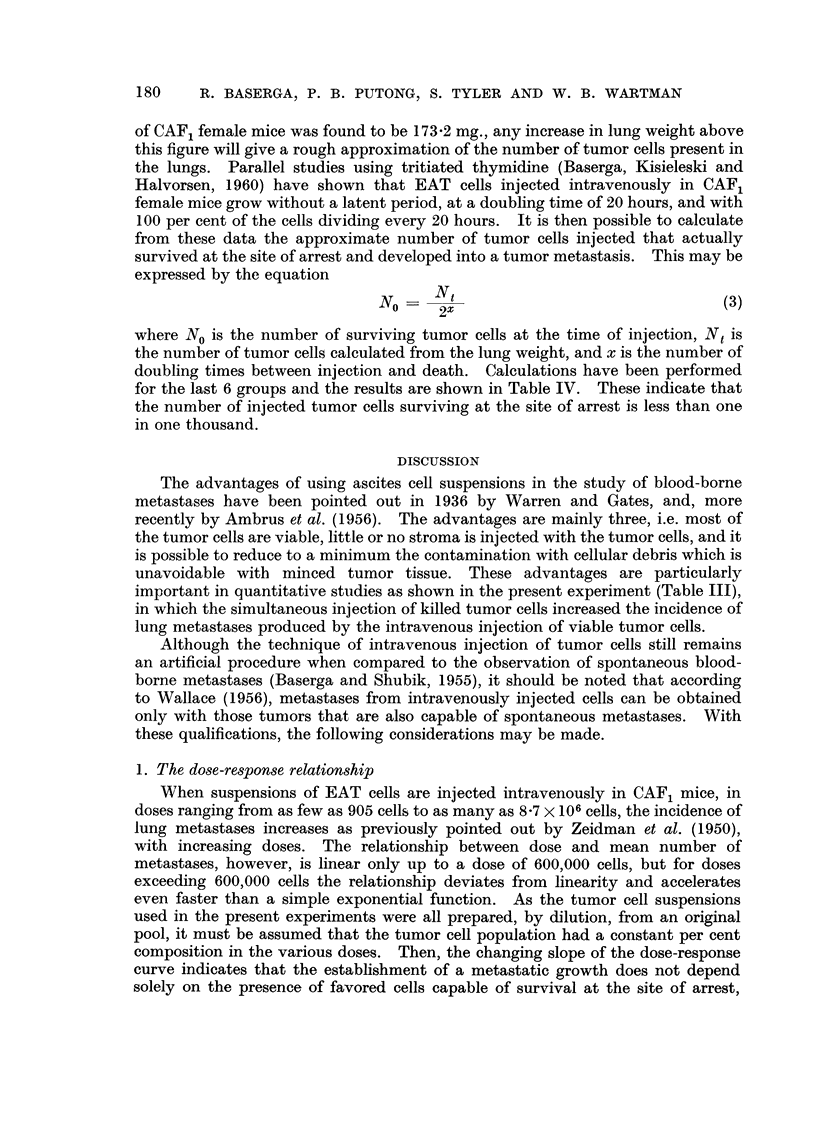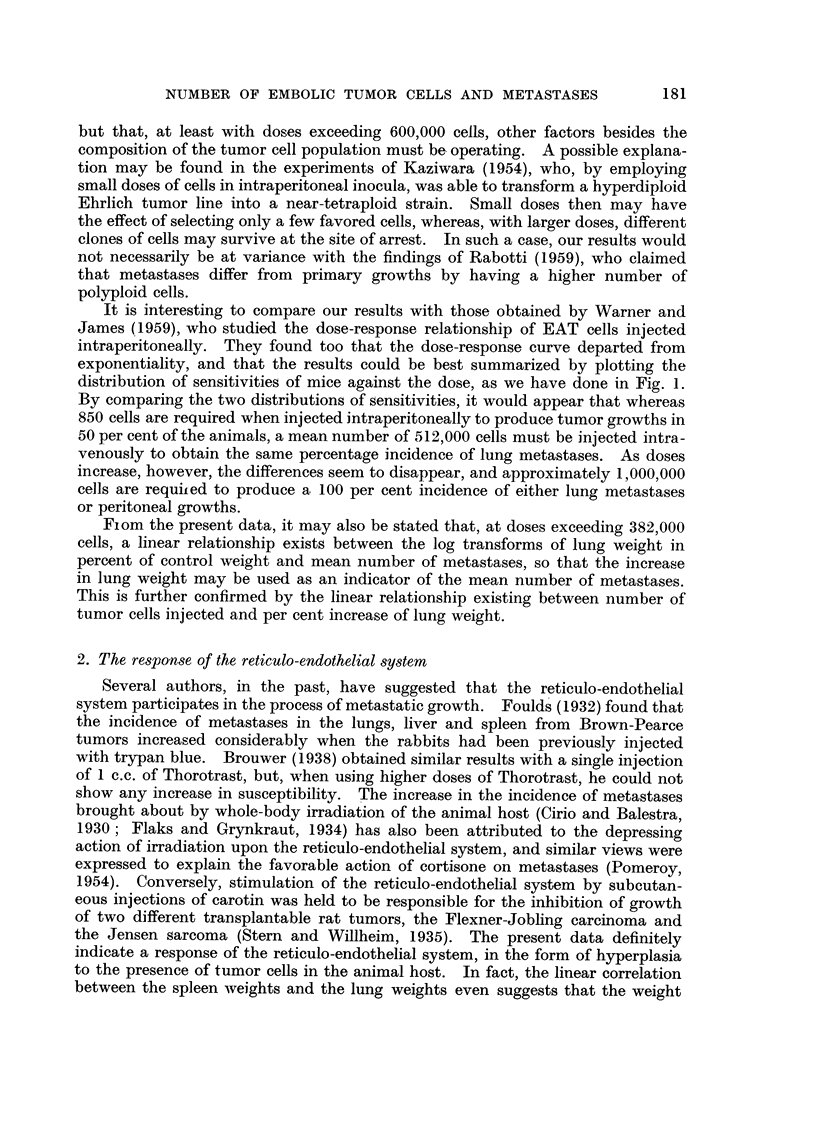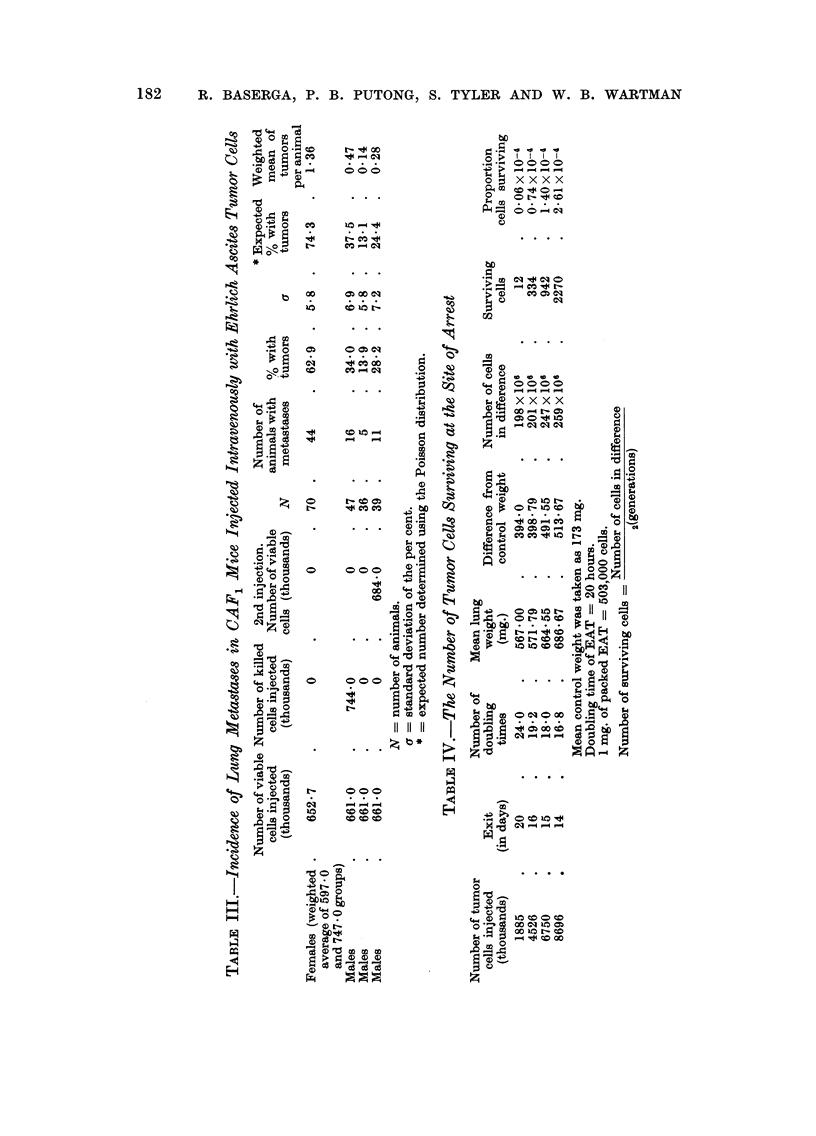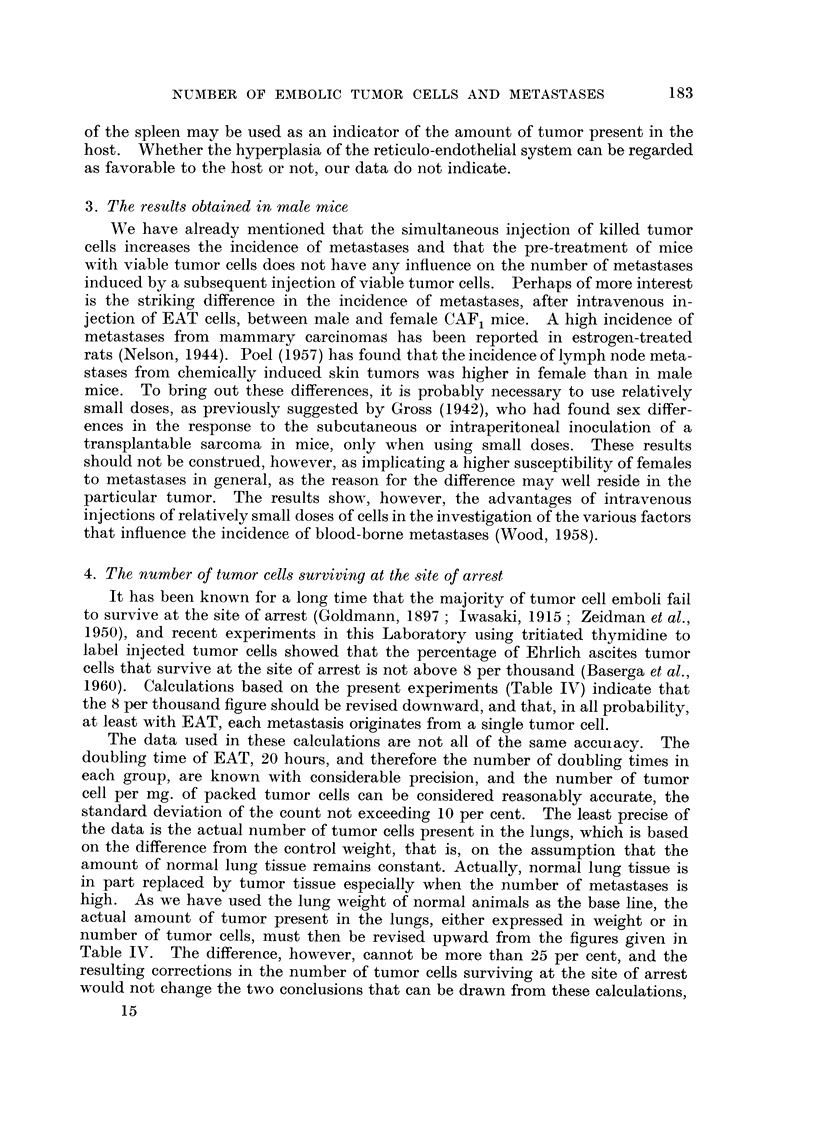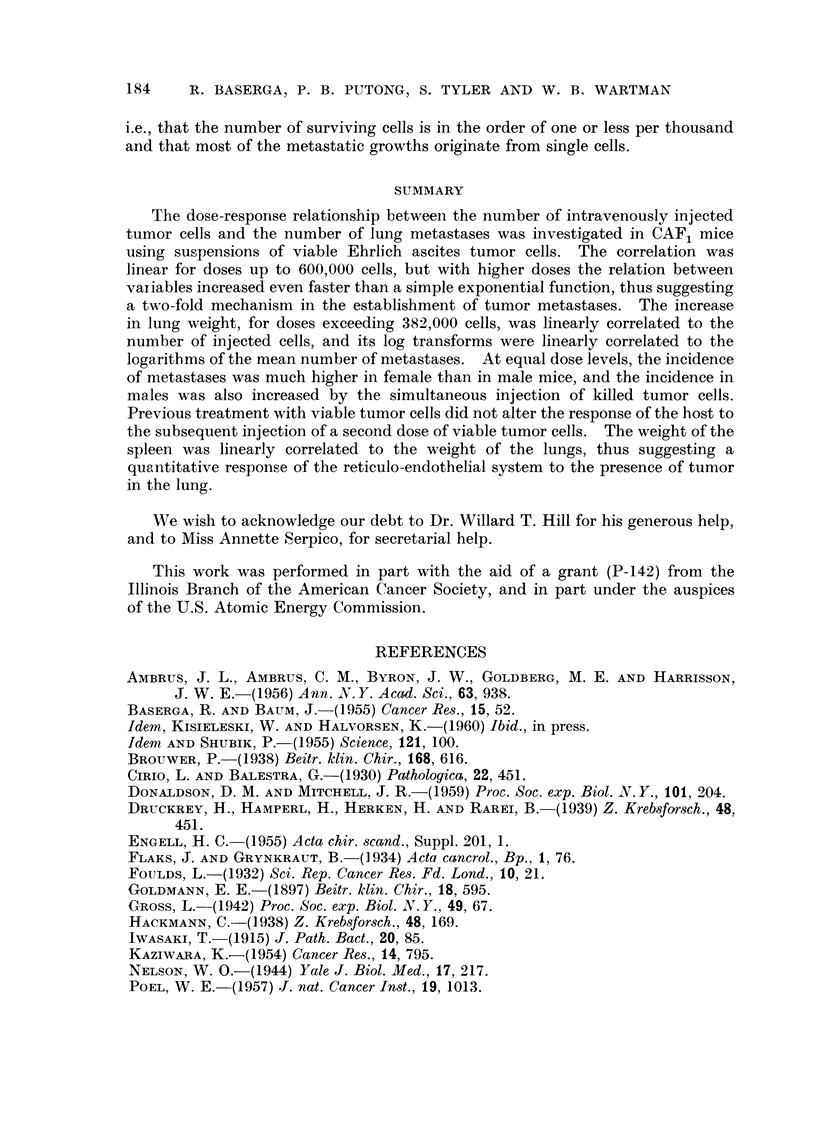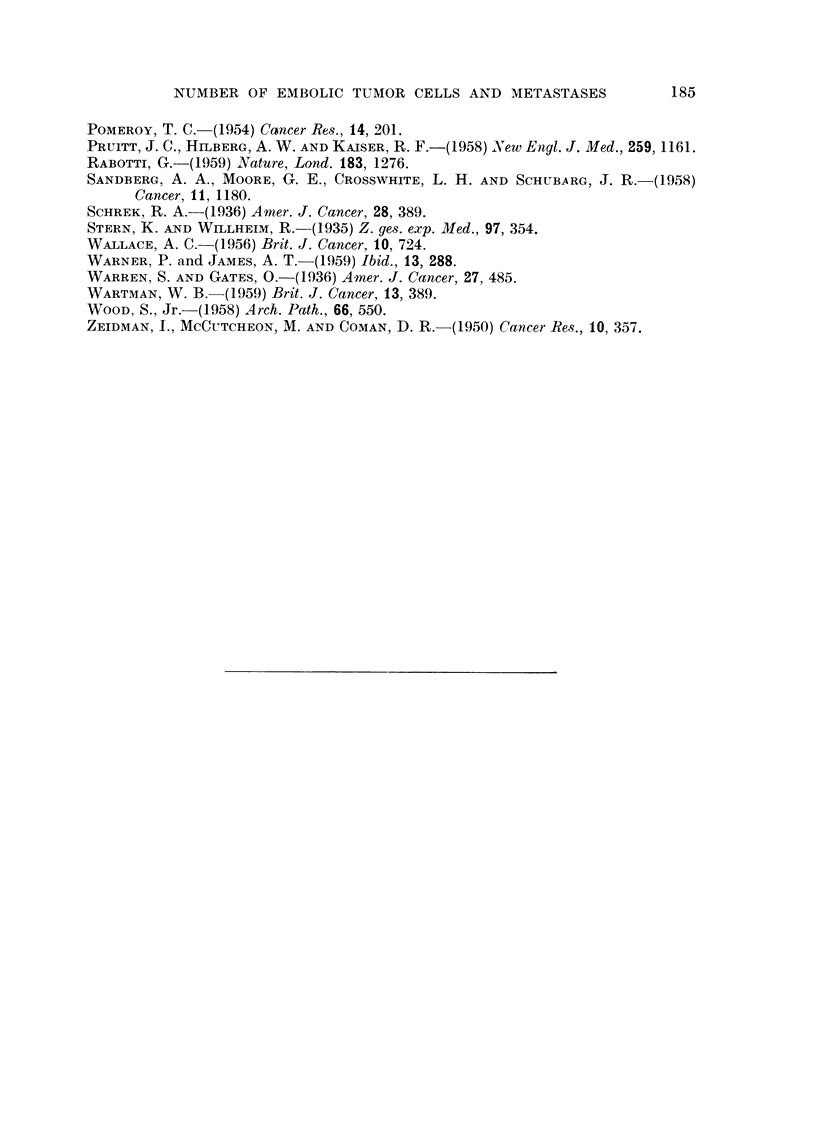# The Dose-response Relationship Between the Number of Embolic Tumor Cells and the Incidence of Blood-borne Metastases

**DOI:** 10.1038/bjc.1960.21

**Published:** 1960-06

**Authors:** R. Baserga, P. B. Putong, S. Tyler, W. B. Wartman


### THE DOSE-RESPONSE RELATIONSHIP BETWEEN THE NUMBER OF EMBOLIC TUMOR CELLS AND THE INCIDENCE OF BLOOD-BORNE METASTASES

R. BASERGA, P. B. PUTONG, S. TYLER AND W. B. WARTMAN

*From the Department of Pathology of Northwestern University Medical School, Chicago, and the Division of Biological and Medical Research, The Argonne National Laboratory, Lemont, Illinois, U.S.A.*

Received for publication April 16, 1960

It is known that the incidence of blood-borne tumour metastases may be influenced by many factors and by several experimental procedures (Baserga and Baum, 1955 ; Wood, 1958). One of these determining factors is the number of embolic tumor cells circulating in the blood stream. Zeidman, McCutcheon and Coman (1950) showed that the number of lung metastases in mice was roughly proportional to the number of tumor cells injected intravenously. More recently, the frequent finding of tumor cells in random samples of venous blood from tumor-bearing patients (Engell, 1955 ; Sandbert *et al.*, 1958 ; Pruitt, Hilberg and Kaiser, 1958) has indicated that a relatively large number of tumour cells may actually be present at any given time in the blood stream of these patients. These observations have prompted us to expand the investigation of Zeidman and co-workers to cover a wider range of the dose-response curve, with the objective of establishing a quantitative relationship between the number of embolic tumour cells and the incidence of metastases. Because of the quantitative conditions of the present experiment we thought it worthwhile to investigate at the same time other factors that have been said to affect the incidence of blood-borne metastases, such as the sex of the animal (Poel, 1957), the simultaneous injection of killed tumor cells (Donaldson and Mitchell, 1959) or the pre-treatment with viable tumor cells (Hackmann, 1938) as well as the response of the reticulo-endothelial system to the presence of metastases (Foulds, 1932 ; Druckrey *et al.*, 1939 ; Wartman, 1959). For these purposes, different doses of viable Ehrlich ascites tumor cells were injected into the tail vein of mice of both sexes, two groups being used to study the effects of the simultaneous injection of killed tumor cells or previous injection of viable tumor cells. The incidence and number of lung metastases in each group was determined by actual count, and the weights of the lungs, spleen, liver and kidneys were used to establish a quantitative index of the response of the reticulo-endothelial system to the presence of tumor metastases.

### MATERIALS AND METHODS

$C \times AF_1$ mice, of both sexes and 4–6 months old which had been bred in the Department of Pathology of Northwestern University Medical School by Dr. Willard T. Hill, were kept in plastic cages in air-conditioned quarters, and given Rockland mouse diet and water *ad libitum.*

The tumor was Ehrlich ascites tumor, a subline of which has been propagated in this Laboratory for 5 years by weekly intraperitoneal injections to healthy carriers. Suspensions of viable tumor cells were prepared as follows: the peritoneal fluid was aspirated 7–10 days after inoculation and centrifuged at 3000 r.p.m. for 10 minutes, the supernatant discarded and the tumor cells resuspended in sterile isotonic saline in the desired dilution. The tumour cells were counted in a hemocytometer, 5 to 10 counts being used for each dilution. Due to the difficulties involved in obtaining round numbers of tumor cells, suspensions that were as near the desired dose level as possible were used. The number of viable cells in the suspensions as determined by Schreck's method (1936), ranged between 93 and 98 per cent.

Suspensions of non-viable tumor cells were prepared as follows: 7–10-days-old Ehrlich ascites tumor was aspirated from the peritoneal cavity of healthy carriers, placed in glass tubes, centrifuged at 3000 r.p.m. for 10 minutes and the supernatant discarded. Ten per cent buffered formalin was added to the packed tumor cells in a ratio of 7 : 1 and the suspension was placed in a refrigerator at 4° C. for 12–18 hours. The formalinized cells were then centrifuged and washed 4 times with normal saline solution and finally resuspended in sterile isotonic saline in the desired dilution. Viability counts showed 100 per cent non-viable cells.

Only female mice were used to establish the dose-response curve. The number of tumor cells injected and the number of animals in each group are shown in Table I. For the second part of the experiment, on the incidence of metastases

TABLE I.—*Incidence of Metastases in $CAF_1$ Female Mice Injected Intravenously with Ehrlich Ascites Tumor Cells*

| Number of tumor cells injected ± $S$ | $N$ | % of mice with metastases | Number of metastases | Number of metastases per mouse |
|---|---|---|---|---|
| None | 25 | 0·0 | 0 | 0·000 |
| 905±170 | 19 } 27 | 10·5 } 7·4 | 2 } 2 | 0·105 } 0·074 |
| 14,390±700 | 8 | 0·0 | 0 | 0·000 |
| 93,200±6,800 | 16 | 25·0 | 4 | 0·250 |
| 382,000±80,000 | 18 | 33·3 | 8 | 0·444 |
| 597,000±90,000 | 44 | 52·3 | 37 | 0·864 |
| 747,000±110,000 | 26 | 80·8 | 58 | 2·192 |
| 928,000±76,000 | 16 | 100·0 | 97 | 6·062 |
| 1,180,000±88,000 | 8 | 100·0 | 114 | 14·250 |
| 1,654,000±140,000 | 20 | 100·0 | 815 | 40·750 |
| 1,885,000±350,000 | 10 | 100·0 | — | >200 |
| 4,526,000±180,000 | 14 | 100·0 | — | >200 |
| 6,750,000±120,000 | 11 | 100·0 | — | >200 |
| 8,696,000±430,000 | 6 | 100·0 | — | >200 |

$S$ = standard deviation.
$N$ = number of mice.

in mice previously treated with viable or non-viable tumor cells, only male mice were used. The number of tumor cells injected and the number of animals in each group are shown in Table III. All injections, either of viable or non-viable cells, were made into the tail vein, using a 27-gauge needle and a calibrated syringe. About half the injections resulted in local growths at the injection site in the tail or at the root of the tail. All animals that showed the slightest evidence of local growths were discarded, and were not included in the computations.

Except when otherwise stated, the mice were sacrificed by cervical dislocation 30 days after the injection of tumor cells. The body weight and the weights of the lungs, liver, spleen and left kidney were determined for each animal. The lungs were examined and the number of grossly visible metastases counted by two different observers. Precise counts were not possible when the number of metastases in both lungs was above 200.

The number of tumor cells in a given weight of packed Ehrlich ascites tumor was determined as follows : 5 ml. of tumor cell suspension, from 8-day-old peritoneal growths were measured in a calibrated pipette and the number of cells per ml. was determined as usual with a hemocytometer. After centrifuging and discarding the supernatant, the packed tumor cells were weighed on an analytical balance, the weight obtained being taken as the weight of the number of cells contained in 5 ml. of tumor suspension. The procedure was repeated on 5 different animals, and the results were averaged.

### RESULTS

### 1. *Dose-response relationships*

Table I shows the incidence of lung metastases in $CAF_1$ female mice following intravenous injection of Ehrlich ascites tumor cells. Animals alive on the 30th day of the experiment were sacrificed. Other animals were autopsied on the day of death. All animals of the groups receiving less than 1,180,000 cells were alive on the 30th day, and the last 5 groups had mean survival times equal to 26, 20, 16, 15 and 14 days respectively. The incidence of metastases below 100 per cent when plotted on probability paper, was linearly related to dose (Fig. 1). This indicates that the distribution of susceptibilities to Ehrlich ascites tumor cells is approximately normal with least square estimates of mean and standard deviation equal to 512,000 and 394,000 cells respectively. The relationship between average number of metastases per mouse and dose, for groups in which a tumor count could be made, is shown in Fig. 2 and is definitely nonlinear. However, the difference between the trend seen at small doses and that characterizing large dose groups suggests that at least two processes may influence the pattern seen in Fig. 2. It is of interest to note that for doses equal to or less than 600,000 cells, the relation between variables is essentially linear ; while for doses exceeding 600,000 cells the pattern of points accelerates even faster than a simple exponential function. Also for doses not exceeding 600,000 cells the group incidence predicted, based on Poisson expectations with the observed number of metastases per mouse as mean value, agrees closely with the observed incidence within groups. On the contrary, for doses above 600,000 cells, the expectations greatly deviate from the observed.

Since the lung is the principal site of establishment and growth of Ehrlich ascites tumor cells injected intravenously, a weight change in this organ should reflect the severity of the insult sustained by the organism as the dose is increased and provide a means of interpolation between the experimentally controlled dosages. The increase in lung weight with injected dose (Table II) was found to be a moderately sensitive indicator. Since the average weight of the lungs for groups injected with 382,000 cells or less seemed to vary about the mean of the controls in a random fashion, the mean weight of the lungs of the controls was taken as the weighted average of these groups and was found to be 173·2 mg. A linear relationship between mean lung weight as per cent of the control value and dose exists over a range of doses extending from 600,000 cells to approximately

2 million cells (Fig. 3). Lung weights for injected doses above this range tend toward an asymptotic or probably an anatomical limiting value which is in the neighborhood of 700 mg. Within this range of doses, the magnitude of the slope of the least square line indicates that the lung weight increases at a constant rate of 0·165 grams per 1000 cells injected. The equation of the least square line is :

$$L = 3{\cdot}55 + 0{\cdot}165D, \qquad 6 \times 10^2 D\ 2 \times 10^3 \tag{1}$$

Where $L$ is the mean lung weight in per cent of the control value and $D$ is the dose in thousands of injected cells.

Fig. 1.—Probit transformation of the dose-incidence curve. Per cent of animals with lung metastases, $CAF_1$ female mice injected intravenously with Ehrlich ascites tumor cells. Slope = 0·002534 ± 0·000498 ; $ED_{50}$ = 512·9 ± 51·2 × $10^3$ cells.

Although the dependence between lung weight and mean number of metastases per animal does not allow a simple explanation, an empirically derived functional relationship between these variables is presented in Fig. 4. A log-log plot of the variables shows a linear relationship between their log transforms. This relationship at doses exceeding 382,000 cells is expressed by the power law

$$M = 3{\cdot}689(10^{-10})\, L^{4{\cdot}622} \tag{2}$$

where $M$ is the mean number of metastases per animal and $L$ is the mean lung weight in per cent of the control weight. From equations (1) and (2), an empirical expression of the dependence between number of metastases and dose can readily be determined.

FIG. 2.—Relationship between average number of lung metastases per mouse and number of tumor cells injected. $CAF_1$ female mice injected intravenously with Ehrlich ascites tumor cells.

TABLE II.—*Mean Weights of Lungs, Spleen, Liver and Left Kidney of $CAF_1$ Female Mice Injected Intravenously with Ehrlich Ascites Tumor Cells*

| Number of tumor cells injected | Body weight | | | Lungs weight | | | Liver weight | | | Spleen weight | | | Kidney weight | | |
|---|---|---|---|---|---|---|---|---|---|---|---|---|---|---|---|
| | $n$ | $\bar{X}$ | $S\bar{x}$ | $n$ | $\bar{X}$ | $S\bar{x}$ | $n$ | $\bar{X}$ | $S\bar{x}$ | $n$ | $\bar{X}$ | $S\bar{x}$ | $n$ | $\bar{X}$ | $S\bar{x}$ |
| None | 25 | 26·8 | 0·3 | 25 | 182 | 2 | 14 | 1529 | 61 | 25 | 127 | 4 | 25 | 174 | 2 |
| 905 | 19 | 25·9 | 0·5 | 19 | 181 | 4 | 19 | 1301 | 29 | 19 | 132 | 3 | 19 | 165 | 4 |
| 14,390 | 8 | 25·8 | 1·2 | 8 | 161 | 4 | 8 | 1334 | 33 | 8 | 124 | 7 | 8 | 152 | 4 |
| 93,200 | 16 | 27·9 | 0·4 | 16 | 178 | 5 | 8 | 1472 | 43 | 16 | 159 | 7 | 16 | 173 | 3 |
| 382,000 | 18 | 26·0 | 0·3 | 18 | 154 | 4 | 18 | 1274 | 38 | 18 | 125 | 12 | 18 | 159 | 3 |
| 597,000 | 44 | 26·5 | 0·3 | 44 | 182 | 4 | 16 | 1539 | 80 | 42 | 158 | 6 | 23 | 181 | 4 |
| 747,000 | 25 | 27·1 | 0·6 | 26 | 225 | 11 | 9 | 1485 | 65 | 23 | 229 | 27 | 10 | 191 | 5 |
| 928,000 | 16 | 26·2 | 0·5 | 16 | 277 | 11 | — | — | — | 16 | 224 | 12 | 10 | 185 | 3 |
| 1,180,000 | 8 | 24·6 | 1·6 | 8 | 344 | 41 | — | — | — | 7 | 241 | 23 | — | — | — |
| 1,654,000 | 20 | 25·6 | 0·4 | 10 | 451 | 17 | 17 | 1656 | 41 | 19 | 327 | 12 | 19 | 183 | 4 |
| 1,885,000 | 10 | 25·6 | 1·0 | 10 | 567 | 36 | — | — | — | 6 | 338 | 40 | — | — | — |
| 4,526,000 | 14 | 24·7 | 0·8 | 14 | 572 | 34 | 6 | 1895 | 46 | 6 | 361 | 14 | 6 | 175 | 7 |
| 6,750,000 | 11 | 25·1 | 0·8 | 11 | 665 | 36 | — | — | — | — | — | — | — | — | — |
| 8,696,000 | 6 | 25·6 | 0·9 | 6 | 687 | 48 | — | — | — | 3 | 327 | 12 | — | — | — |
| Subq. injection | 14 | 26·9 | 0·5 | 14 | 205 | 7 | 2 | 1925 | 145 | 22 | 504 | 23 | 4 | 189 | 12 |

$n$ = number of measures included in mean.
$\bar{X}$ = mean, in g. for body weight, in mg. for lungs, spleen, liver and kidney.
$S\bar{x}$ = standard error of the mean.
Subq. injection : a group of mice with huge tumor growths at the root of the tail.

2. *Response of the reticulo-endothelial system*

Even though not a single metastasis was found, either grossly or histologically, in any organ except the lung, a significant response to the presence of tumor cells was noted in the spleen. The weight of the spleen highly correlates with number of tumor cells injected (Table II). In the liver the correlation is partially obscured by variations related to the body weight while in the kidney, a statistically significant trend between weight and dose is not present in the sample. Spleen weight and dose are linearly related (Fig. 3), and thus, by equation 1,

FIG. 3.—Lung and spleen weights versus dose in $CAF_1$ female mice injected intravenously with Ehrlich ascites tumor cells. Weight of control lungs : 173·2 mg.; slope = 0·1651 ± 0·0094, intercept 3·55 ± 11·88. Weight of control spleen : 133·6 mg.; slope = 0·0962 ± 0·0137, intercept = 77·28 ± 17·17.

a straight line relationship between the weights of the spleen and lungs is implied. In those mice in which an improper intravenous injection resulted in a huge growth at the root of the tail (Table II), the weight of the spleen was, on the average, 3·7 times the weight of controls. This indicates that the spleen may respond to the presence of tumor cells regardless of the site of tumor growth.

3. *The results in male mice*

These are shown in Table III, from which it is apparent that the incidence of metastases in male mice is considerably lower than in female mice, the 5 per cent critical level being used as a measure of significance. It will also be noted that when killed tumor cells are injected simultaneously with viable cells, the incidence of lung metastases in male mice increases.

When male mice, are injected, 80 days apart, with two similar doses of viable tumor cells, the incidence of lung metastases is about twice that observed in mice injected with a single dose. This seems to indicate that previous treatment with viable tumor cells does not alter the response of the host to a second injection of viable tumor cells.

FIG. 4.—Log-log plot of lung weight versus mean number of metastases in $CAF_1$ female mice injected intravenously with Ehrlich ascites tumor cells. Slope = 4·622 ± 0·208, intercept = − 9·433 ± 0·471.

4. *The estimated number of tumor cells surviving at the site of arrest*

With the procedure outlined in Methods and Materials, it was found that 1 ml. of EAT contained on the average, 139,84 × $10^6$ cells, and that after discarding the supernatant, this number of packed cells weighed 278 mg. (mean of 5 trials). This meant that 1 mg. of packed EAT cells contained an average of 503,000 cells. Determinations of the percentage dry weight of the lungs showed that there was little difference between the control lungs (19 per cent dry weight) and the heaviest lungs filled with tumor (18 per cent dry weight). As the mean weight of the lungs of $CAF_1$ female mice was found to be 173·2 mg., any increase in lung weight above this figure will give a rough approximation of the number of tumor cells present in the lungs. Parallel studies using tritiated thymidine (Baserga, Kisieleski and Halvorsen, 1960) have shown that EAT cells injected intravenously in $CAF_1$ female mice grow without a latent period, at a doubling time of 20 hours, and with 100 per cent of the cells dividing every 20 hours. It is then possible to calculate from these data the approximate number of tumor cells injected that actually survived at the site of arrest and developed into a tumor metastasis. This may be expressed by the equation

$$N_0 = \frac{N_t}{2^x} \quad (3)$$

where $N_0$ is the number of surviving tumor cells at the time of injection, $N_t$ is the number of tumor cells calculated from the lung weight, and $x$ is the number of doubling times between injection and death. Calculations have been performed for the last 6 groups and the results are shown in Table IV. These indicate that the number of injected tumor cells surviving at the site of arrest is less than one in one thousand.

### DISCUSSION

The advantages of using ascites cell suspensions in the study of blood-borne metastases have been pointed out in 1936 by Warren and Gates, and, more recently by Ambrus *et al.* (1956). The advantages are mainly three, i.e. most of the tumor cells are viable, little or no stroma is injected with the tumor cells, and it is possible to reduce to a minimum the contamination with cellular debris which is unavoidable with minced tumor tissue. These advantages are particularly important in quantitative studies as shown in the present experiment (Table III), in which the simultaneous injection of killed tumor cells increased the incidence of lung metastases produced by the intravenous injection of viable tumor cells.

Although the technique of intravenous injection of tumor cells still remains an artificial procedure when compared to the observation of spontaneous blood-borne metastases (Baserga and Shubik, 1955), it should be noted that according to Wallace (1956), metastases from intravenously injected cells can be obtained only with those tumors that are also capable of spontaneous metastases. With these qualifications, the following considerations may be made.

### 1. *The dose-response relationship*

When suspensions of EAT cells are injected intravenously in $CAF_1$ mice, in doses ranging from as few as 905 cells to as many as $8{\cdot}7 \times 10^6$ cells, the incidence of lung metastases increases as previously pointed out by Zeidman *et al.* (1950), with increasing doses. The relationship between dose and mean number of metastases, however, is linear only up to a dose of 600,000 cells, but for doses exceeding 600,000 cells the relationship deviates from linearity and accelerates even faster than a simple exponential function. As the tumor cell suspensions used in the present experiments were all prepared, by dilution, from an original pool, it must be assumed that the tumor cell population had a constant per cent composition in the various doses. Then, the changing slope of the dose-response curve indicates that the establishment of a metastatic growth does not depend solely on the presence of favored cells capable of survival at the site of arrest, but that, at least with doses exceeding 600,000 cells, other factors besides the composition of the tumor cell population must be operating. A possible explanation may be found in the experiments of Kaziwara (1954), who, by employing small doses of cells in intraperitoneal inocula, was able to transform a hyperdiploid Ehrlich tumor line into a near-tetraploid strain. Small doses then may have the effect of selecting only a few favored cells, whereas, with larger doses, different clones of cells may survive at the site of arrest. In such a case, our results would not necessarily be at variance with the findings of Rabotti (1959), who claimed that metastases differ from primary growths by having a higher number of polyploid cells.

It is interesting to compare our results with those obtained by Warner and James (1959), who studied the dose-response relationship of EAT cells injected intraperitoneally. They found too that the dose-response curve departed from exponentiality, and that the results could be best summarized by plotting the distribution of sensitivities of mice against the dose, as we have done in Fig. 1. By comparing the two distributions of sensitivities, it would appear that whereas 850 cells are required when injected intraperitoneally to produce tumor growths in 50 per cent of the animals, a mean number of 512,000 cells must be injected intravenously to obtain the same percentage incidence of lung metastases. As doses increase, however, the differences seem to disappear, and approximately 1,000,000 cells are required to produce a 100 per cent incidence of either lung metastases or peritoneal growths.

From the present data, it may also be stated that, at doses exceeding 382,000 cells, a linear relationship exists between the log transforms of lung weight in percent of control weight and mean number of metastases, so that the increase in lung weight may be used as an indicator of the mean number of metastases. This is further confirmed by the linear relationship existing between number of tumor cells injected and per cent increase of lung weight.

2. *The response of the reticulo-endothelial system*

Several authors, in the past, have suggested that the reticulo-endothelial system participates in the process of metastatic growth. Foulds (1932) found that the incidence of metastases in the lungs, liver and spleen from Brown-Pearce tumors increased considerably when the rabbits had been previously injected with trypan blue. Brouwer (1938) obtained similar results with a single injection of 1 c.c. of Thorotrast, but, when using higher doses of Thorotrast, he could not show any increase in susceptibility. The increase in the incidence of metastases brought about by whole-body irradiation of the animal host (Cirio and Balestra, 1930; Flaks and Grynkraut, 1934) has also been attributed to the depressing action of irradiation upon the reticulo-endothelial system, and similar views were expressed to explain the favorable action of cortisone on metastases (Pomeroy, 1954). Conversely, stimulation of the reticulo-endothelial system by subcutaneous injections of carotin was held to be responsible for the inhibition of growth of two different transplantable rat tumors, the Flexner-Jobling carcinoma and the Jensen sarcoma (Stern and Willheim, 1935). The present data definitely indicate a response of the reticulo-endothelial system, in the form of hyperplasia to the presence of tumor cells in the animal host. In fact, the linear correlation between the spleen weights and the lung weights even suggests that the weight

TABLE III.—*Incidence of Lung Metastases in $CAF_1$ Mice Injected Intravenously with Ehrlich Ascites Tumor Cells*

| | Number of viable cells injected (thousands) | Number of killed cells injected (thousands) | 2nd injection. Number of viable cells (thousands) | $N$ | Number of animals with metastases | % with tumors | $\sigma$ | * Expected % with tumors | Weighted mean of tumors per animal |
|---|---|---|---|---|---|---|---|---|---|
| Females (weighted average of 597·0 and 747·0 groups) | 652·7 | 0 | 0 | 70 | 44 | 62·9 | 5·8 | 74·3 | 1·36 |
| Males | 661·0 | 744·0 | 0 | 47 | 16 | 34·0 | 6·9 | 37·5 | 0·47 |
| Males | 661·0 | 0 | 0 | 36 | 5 | 13·9 | 5·8 | 13·1 | 0·14 |
| Males | 661·0 | 0 | 684·0 | 39 | 11 | 28·2 | 7·2 | 24·4 | 0·28 |

$N$ = number of animals.

$\sigma$ = standard deviation of the per cent.

TABLE IV.—*The Number of Tumor Cells Surviving at the Site of Arrest*

| Number of tumor cells injected (thousands) | Exit (in days) | Number of doubling times | Mean lung weight (mg.) | Difference from control weight | Number of cells in difference | Surviving cells | Proportion cells surviving |
|---|---|---|---|---|---|---|---|
| 1885 | 20 | 24·0 | 567·00 | 394·0 | $198 \times 10^6$ | 12 | $0{\cdot}06 \times 10^{-4}$ |
| 4526 | 16 | 19·2 | 571·79 | 398·79 | $201 \times 10^6$ | 334 | $0{\cdot}74 \times 10^{-4}$ |
| 6750 | 15 | 18·0 | 664·55 | 491·55 | $247 \times 10^6$ | 942 | $1{\cdot}40 \times 10^{-4}$ |
| 8696 | 14 | 16·8 | 686·67 | 513·67 | $259 \times 10^6$ | 2270 | $2{\cdot}61 \times 10^{-4}$ |

Mean control weight was taken as 173 mg.

Doubling time of EAT = 20 hours.

1 mg. of packed EAT = 503,000 cells.

$$\text{Number of surviving cells} = \frac{\text{Number of cells in difference}}{_2(\text{generations})}$$

of the spleen may be used as an indicator of the amount of tumor present in the host. Whether the hyperplasia of the reticulo-endothelial system can be regarded as favorable to the host or not, our data do not indicate.

3. *The results obtained in male mice*

We have already mentioned that the simultaneous injection of killed tumor cells increases the incidence of metastases and that the pre-treatment of mice with viable tumor cells does not have any influence on the number of metastases induced by a subsequent injection of viable tumor cells. Perhaps of more interest is the striking difference in the incidence of metastases, after intravenous injection of EAT cells, between male and female $CAF_1$ mice. A high incidence of metastases from mammary carcinomas has been reported in estrogen-treated rats (Nelson, 1944). Poel (1957) has found that the incidence of lymph node metastases from chemically induced skin tumors was higher in female than in male mice. To bring out these differences, it is probably necessary to use relatively small doses, as previously suggested by Gross (1942), who had found sex differences in the response to the subcutaneous or intraperitoneal inoculation of a transplantable sarcoma in mice, only when using small doses. These results should not be construed, however, as implicating a higher susceptibility of females to metastases in general, as the reason for the difference may well reside in the particular tumor. The results show, however, the advantages of intravenous injections of relatively small doses of cells in the investigation of the various factors that influence the incidence of blood-borne metastases (Wood, 1958).

4. *The number of tumor cells surviving at the site of arrest*

It has been known for a long time that the majority of tumor cell emboli fail to survive at the site of arrest (Goldmann, 1897 ; Iwasaki, 1915 ; Zeidman *et al.*, 1950), and recent experiments in this Laboratory using tritiated thymidine to label injected tumor cells showed that the percentage of Ehrlich ascites tumor cells that survive at the site of arrest is not above 8 per thousand (Baserga *et al.*, 1960). Calculations based on the present experiments (Table IV) indicate that the 8 per thousand figure should be revised downward, and that, in all probability, at least with EAT, each metastasis originates from a single tumor cell.

The data used in these calculations are not all of the same accuiacy. The doubling time of EAT, 20 hours, and therefore the number of doubling times in each group, are known with considerable precision, and the number of tumor cell per mg. of packed tumor cells can be considered reasonably accurate, the standard deviation of the count not exceeding 10 per cent. The least precise of the data is the actual number of tumor cells present in the lungs, which is based on the difference from the control weight, that is, on the assumption that the amount of normal lung tissue remains constant. Actually, normal lung tissue is in part replaced by tumor tissue especially when the number of metastases is high. As we have used the lung weight of normal animals as the base line, the actual amount of tumor present in the lungs, either expressed in weight or in number of tumor cells, must then be revised upward from the figures given in Table IV. The difference, however, cannot be more than 25 per cent, and the resulting corrections in the number of tumor cells surviving at the site of arrest would not change the two conclusions that can be drawn from these calculations, i.e., that the number of surviving cells is in the order of one or less per thousand and that most of the metastatic growths originate from single cells.

SUMMARY

The dose-response relationship between the number of intravenously injected tumor cells and the number of lung metastases was investigated in $CAF_1$ mice using suspensions of viable Ehrlich ascites tumor cells. The correlation was linear for doses up to 600,000 cells, but with higher doses the relation between variables increased even faster than a simple exponential function, thus suggesting a two-fold mechanism in the establishment of tumor metastases. The increase in lung weight, for doses exceeding 382,000 cells, was linearly correlated to the number of injected cells, and its log transforms were linearly correlated to the logarithms of the mean number of metastases. At equal dose levels, the incidence of metastases was much higher in female than in male mice, and the incidence in males was also increased by the simultaneous injection of killed tumor cells. Previous treatment with viable tumor cells did not alter the response of the host to the subsequent injection of a second dose of viable tumor cells. The weight of the spleen was linearly correlated to the weight of the lungs, thus suggesting a quantitative response of the reticulo-endothelial system to the presence of tumor in the lung.

We wish to acknowledge our debt to Dr. Willard T. Hill for his generous help, and to Miss Annette Serpico, for secretarial help.

This work was performed in part with the aid of a grant (P-142) from the Illinois Branch of the American Cancer Society, and in part under the auspices of the U.S. Atomic Energy Commission.

### REFERENCES

AMBRUS, J. L., AMBRUS, C. M., BYRON, J. W., GOLDBERG, M. E. AND HARRISSON, J. W. E.—(1956) *Ann. N.Y. Acad. Sci.*, **63**, 938.

BASERGA, R. AND BAUM, J.—(1955) *Cancer Res.*, **15**, 52.

*Idem*, KISIELESKI, W. AND HALVORSEN, K.—(1960) *Ibid.*, in press.

*Idem* AND SHUBIK, P.—(1955) *Science*, **121**, 100.

BROUWER, P.—(1938) *Beitr. klin. Chir.*, **168**, 616.

CIRIO, L. AND BALESTRA, G.—(1930) *Pathologica*, **22**, 451.

DONALDSON, D. M. AND MITCHELL, J. R.—(1959) *Proc. Soc. exp. Biol. N.Y.*, **101**, 204.

DRUCKREY, H., HAMPERL, H., HERKEN, H. AND RAREI, B.—(1939) *Z. Krebsforsch.*, **48**, 451.

ENGELL, H. C.—(1955) *Acta chir. scand.*, Suppl. 201, 1.

FLAKS, J. AND GRYNKRAUT, B.—(1934) *Acta cancrol., Bp.*, **1**, 76.

FOULDS, L.—(1932) *Sci. Rep. Cancer Res. Fd. Lond.*, **10**, 21.

GOLDMANN, E. E.—(1897) *Beitr. klin. Chir.*, **18**, 595.

GROSS, L.—(1942) *Proc. Soc. exp. Biol. N.Y.*, **49**, 67.

HACKMANN, C.—(1938) *Z. Krebsforsch.*, **48**, 169.

IWASAKI, T.—(1915) *J. Path. Bact.*, **20**, 85.

KAZIWARA, K.—(1954) *Cancer Res.*, **14**, 795.

NELSON, W. O.—(1944) *Yale J. Biol. Med.*, **17**, 217.

POEL, W. E.—(1957) *J. nat. Cancer Inst.*, **19**, 1013.

POMEROY, T. C.—(1954) *Cancer Res.*, **14**, 201.
PRUITT, J. C., HILBERG, A. W. AND KAISER, R. F.—(1958) *New Engl. J. Med.*, **259**, 1161.
RABOTTI, G.—(1959) *Nature, Lond.* **183**, 1276.
SANDBERG, A. A., MOORE, G. E., CROSSWHITE, L. H. AND SCHUBARG, J. R.—(1958) *Cancer*, **11**, 1180.
SCHREK, R. A.—(1936) *Amer. J. Cancer*, **28**, 389.
STERN, K. AND WILLHEIM, R.—(1935) *Z. ges. exp. Med.*, **97**, 354.
WALLACE, A. C.—(1956) *Brit. J. Cancer*, **10**, 724.
WARNER, P. and JAMES, A. T.—(1959) *Ibid.*, **13**, **288**.
WARREN, S. AND GATES, O.—(1936) *Amer. J. Cancer*, **27**, 485.
WARTMAN, W. B.—(1959) *Brit. J. Cancer*, **13**, 389.
WOOD, S., Jr.—(1958) *Arch. Path.*, **66**, 550.
ZEIDMAN, I., MCCUTCHEON, M. AND COMAN, D. R.—(1950) *Cancer Res.*, **10**, 357.